# Protocol for mapping gene essentiality changes in yeast using inducible gene deletion mutants

**DOI:** 10.1016/j.xpro.2026.104723

**Published:** 2026-07-20

**Authors:** Sabine van Schie, Jolanda van Leeuwen

**Affiliations:** 1 Department of Systems Biology, University of Massachusetts Chan Medical School, Worcester, MA 01605, USA; 2 Center for Integrative Genomics, University of Lausanne, Lausanne, Switzerland

**Keywords:** Genetics, Genomics, High Throughput Screening, Microbiology, Model Organisms, Systems biology

## Abstract

Gene essentiality can differ among genetically distinct individuals. In this protocol, we describe a systematic approach for identifying genes that are essential in a laboratory strain of the budding yeast *Saccharomyces cerevisiae* but vary in essentiality across other yeast isolates. This approach relies on a collection of inducible essential gene deletion mutants in the laboratory background. We describe steps for crossing this collection to a yeast isolate of interest, selecting haploid segregant progeny, inducing essential gene loss, and determining viability.

For complete details on the use and execution of this protocol, please refer to Batté et al.^1^

## Highlights

• Procedure to identify context-dependent essential genes in budding yeast isolates
• Steps for preparing compatible budding yeast strains
• Description of an inducible essential gene deletion mutant collection
• Instructions for detecting and validating candidate context-dependent essential genes

## Abstract (editor highlights)

Publisher’s note: Undertaking any experimental protocol requires adherence to local institutional guidelines for laboratory safety and ethics.

## Graphical abstract

**Figure:**
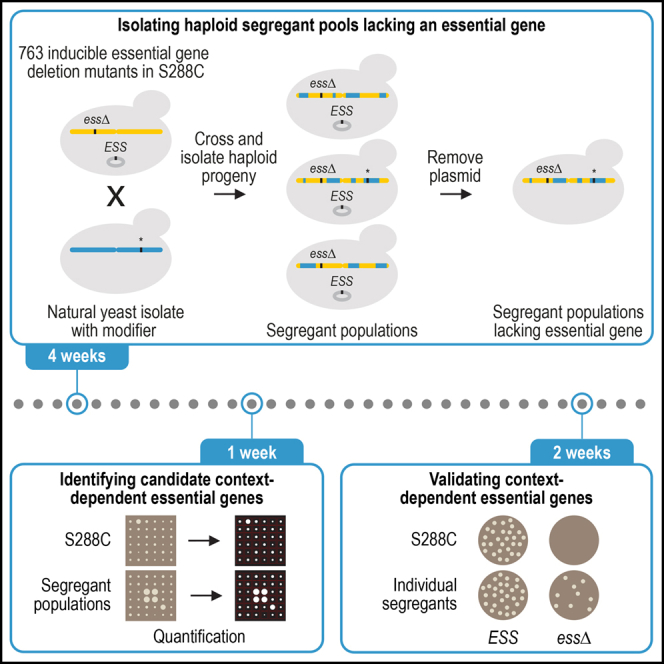

## Before you begin

The effect of a mutation is often influenced by the genetic background in which it occurs. In extreme cases, a mutation can be lethal in one individual while having no effect on viability in another.^1^^,^^2^^,^^3^^,^^4^^,^^5^ Such differences in viability can arise due to modifier variants that bypass the requirement for the essential gene. Identifying genes that vary in essentiality, as well as the underlying genetic causes, increases our understanding of genetic background effects, which is required for the accurate translation of genotypes into phenotypes.

The budding yeast *Saccharomyces cerevisiae* is a powerful model system to study genotype-to-phenotype relationships due to its tractable genetics.^6^ The laboratory strain S288C has ∼6,000 genes, 1,105 of which (∼17%) are required for survival under nutrient-rich growth conditions and are generally considered essential.^7^ Here, we describe a protocol that enables identification of genes that are essential in S288C but vary in essentiality in other budding yeast isolates, based on a collection of inducible gene deletion mutants. In a recent study, we used this approach to investigate the essentiality of ∼800 genes across 18 natural yeast isolates, resulting in the identification of 39 context-dependent essential genes.^1^

**Figure 1:**
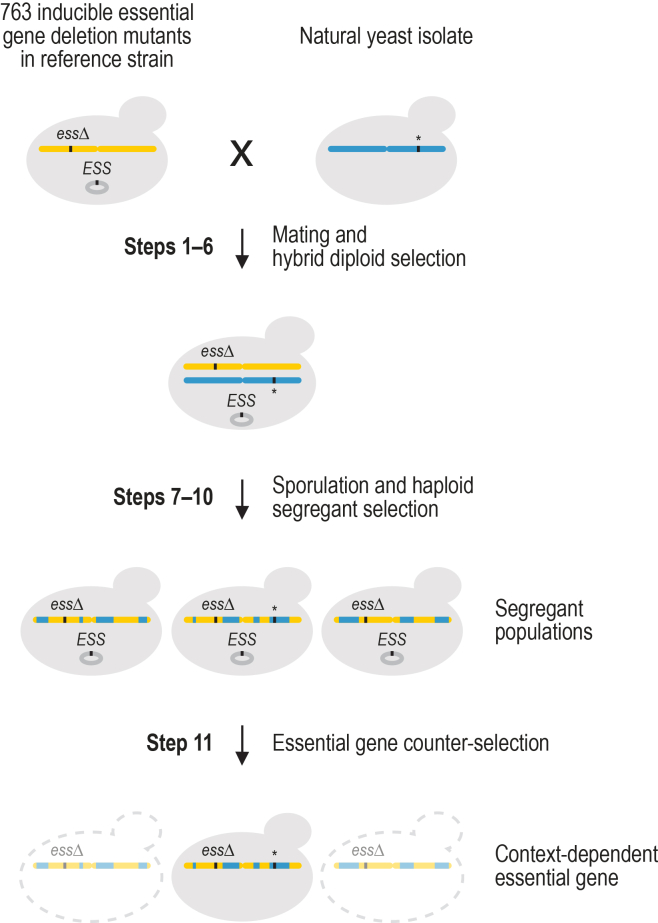
Identifying context-dependent essential genes using inducible gene deletion mutants A collection of haploid strains in the reference background, each deleted for an essential gene in their genome (*essΔ*) but carrying it on a counter-selectable plasmid (*ESS*), is crossed to a natural yeast isolate of interest, as well as an S288C control (steps 1–5). Next, hybrid diploids are isolated on media selecting for the counter-selectable plasmid, the essential gene deletion allele, and the natural yeast isolate marker (step 6). Diploids are driven through meiosis (step 7) and in a series of subsequent selection steps, haploid segregant populations carrying both the deletion allele and the complementing plasmid are isolated (step 8–10). Growth in the absence of the essential gene is subsequently assessed by counter-selecting against the plasmid (step 11). If the natural yeast isolate carried one or more modifier loci (∗) that can bypass the need for the essential gene, growth will be observed in the segregant pool in the absence of the essential gene.

The inducible essential gene deletion collection is derived from the TS-allele-on-plasmid collection^2^ and consists of haploid S288C strains, each deleted for an essential gene in their genome but viable because of the presence of a temperature-sensitive (TS) allele of the essential gene on a counter-selectable plasmid (Figure 1). The essential genes are replaced by either *Kluyveromyces lactis LEU2* (*KlLEU2*) followed by the C-terminal half of *natMX4* or by a nourseothricin resistance cassette (*natR*) followed by the C-terminal half of *kanMX4*. The C-terminal halves of *natMX4* or *kanMX4* can be used to test for unintended integration of the TS allele into the genome, which would typically reconstitute the complete selection cassettes. The TS phenotype is not relevant for the current protocol, and most steps with strains carrying the plasmid are performed at 26°C to ensure proper functioning of the essential gene. Counter-selection against the plasmid is achieved by exposing cells to 5-fluoroorotic acid (5-FOA), which is converted into toxic fluorinated nucleotides by Ura3,^8^ encoded on the plasmid, enabling selection against cells carrying a copy of the essential gene. To make this collection compatible with the current protocol, we introduced markers that can be used to isolate haploid progeny after a cross (*can1Δ*, *lyp1Δ*, and either *STE2pr-SpHIS5* or *STE3pr-LEU2*)^9^ and subsequently selected for *MATα* strains.^1^ The resulting collection consists of strains with two different genotypes: *MATα xxxΔ::KlLEU2_natR(Cterm) can1Δ::STE2pr-SpHIS5 lyp1Δ0 hoΔ::natMX4 his3Δ1 leu2Δ0 ura3Δ0* [*xxx-ts_natR(Nterm)*, *AgSTE3pr-hphR*, *URA3*] and *MATα xxxΔ::natR_kanR(Cterm) can1Δ::SpHIS5 lyp1Δ::STE3pr-LEU2 his3Δ1 leu2Δ0 ura3Δ0* [*xxx-ts_kanR(Nterm)*, *AgMFA2pr-hphR*, *URA3*]. The collection consists of 2,539 strains for 763 different essential genes (∼70% of all essential S288C genes), with 348 of these genes represented by multiple strains harboring different TS alleles.

To identify context-dependent essential genes, the collection is crossed to a yeast isolate of interest and an S288C control strain, the resulting hybrids are driven through meiosis, and pools of haploid segregant progeny are selected carrying both the essential gene deletion allele and the plasmid with the essential gene (Figure 1).^1^ Colonies consisting of pools of around 60,000 haploid segregant progeny^10^ are subsequently transferred to media that selects against the plasmid to identify rare cases where growth is observed in the absence of the essential gene (Figure 1). Alternatively, the counter-selection step can be omitted and the effect of the genetic background on the temperature-sensitive phenotype of the essential gene mutant can be assessed instead.^11^ Although any yeast strain of interest can be used in this protocol, we will focus on the use of natural yeast isolates.

### Innovation

Most studies of context-dependent gene essentiality rely on pooled transposon insertion or CRISPR-based approaches.^5^^,^^12^^,^^13^^,^^14^^,^^15^ Instead, this protocol uses an arrayed mutant collection that enables testing each gene individually for essentiality in a genetic background of interest. This results in higher experimental accuracy compared to pooled assays. Furthermore, by isolating segregant pools for each tested gene, our method facilitates precise mapping of the variants responsible for differential essentiality through bulk segregant analysis.^1^^,^^16^ This allows moving beyond the identification of differences in gene essentiality, to understanding how such differences arise.^1^

### Deleting auxotrophic marker genes in yeast isolates of interest

**Timing: 3–4 weeks**

Before beginning the protocol, verify that the genotype of the yeast isolate of interest is compatible with the procedure. The *HO* locus, which encodes a site-specific endonuclease that initiates mating-type switching,^17^ needs to be deleted to obtain stable haploid strains. The *URA3*, *LEU2*, and *HIS3* genes are used as selection markers during the procedure and need to be deleted as well. The isolate will also need to express a selection marker that is not present in the inducible gene deletion mutant strains, such as *kanMX4*, to enable diploid selection after crossing the strains (Figure 1). This marker can be introduced at any neutral locus using standard yeast genome editing methods.^18^ In addition, an S288C strain with the same genotype is required as a control. The natural yeast isolates that we use in this protocol have the genotype *MAT****a*** *hoΔ::kanMX4 ura3Δ0 leu2Δ0 his3Δ1.*

Below, we describe a strategy for marker-less deletion of *URA3*, *LEU2*, and *HIS3* based on a previously described CRISPR-Cas9 method.^19^ First, the *ura3Δ0*, *his3Δ1*, and *leu2Δ0* deletion alleles are amplified from the S288C genome by PCR, thereby including ∼400 bp of sequence upstream and downstream of the deleted open reading frames. The PCR fragments are then co-transformed with a plasmid expressing Cas9 and a guide RNA (gRNA) targeting the auxotrophic marker gene, into the yeast isolate of interest. Cas9 will create a double-stranded DNA break within the targeted gene, which will promote integration of the PCR product into the genome and replace the gene with the deletion allele.

1. Amplify the deletion alleles from the genome of BY4741 (*MAT****a*** *his3Δ1 leu2Δ0 ura3Δ0 met15Δ0*).
  a. Prepare cell lysates:
    i. Add 20 μL of TE buffer (10 mM Tris-HCl, 1 mM EDTA, pH 8.0) supplemented with 0.1% Triton X-100 to a PCR tube.
    ii. Scrape about one-tenth of a yeast colony (∼250,000 cells) from an agar plate and add to the PCR tube. Mix well.
      ***Note:*** Using too many cells can inhibit the PCR reaction, whereas using too few cells can result in weak or undetectable amplification.
    iii. Incubate for 5 min at 99°C, hold at 10°C.
    iv. Mix and use the suspension as a PCR template.
  b. Prepare a 25-μL PCR reaction (Table 1).
    ***Note:*** Primer sequences for amplifying *his3Δ1, leu2Δ0,* and *ura3Δ0* are listed in the key resources table.
    **Alternatives:** Template DNA from another yeast strain carrying loss-of-function alleles of these genes can be used.
    **Alternatives:** Instead of colony PCR, ∼50 ng of purified genomic DNA per 25-μL PCR reaction can be used as a template.
  c. Run the PCR reaction (Table 2).
  d. Run 5 μL of PCR product on a 1% agarose gel to check for the presence of bands.
2. Co-transform the PCR product and a plasmid containing *Cas9* and a gRNA targeting the auxotrophic marker gene.
  ***Note:*** Delete *URA3*, *HIS3*, and *LEU2* sequentially. Because the gRNA plasmids targeting *HIS3* and *LEU2* carry the *URA3* selection marker, the endogenous *URA3* locus must be deleted first before subsequent deletion of *HIS3* or *LEU2*.
  ***Note:*** Guide RNA plasmids are listed in the key resources table. For each gene, two plasmids carrying different gRNAs targeting that gene are provided. If a gRNA targets a sequence containing a SNP in the natural isolate’s genome, editing efficiency may be reduced. When the genome sequence of the natural isolate is unknown, co-transforming both plasmids targeting the same gene can enhance the likelihood of successful genome editing.
  a. Inoculate 3 mL of YPD with your yeast strain of interest and grow overnight (12–16 h) at 30°C with shaking.
  b. Dilute the overnight culture to OD_600_ = 0.2 in 5 mL of YPD.
  c. Grow for another 3–4 h at 30°C to OD_600_ = 0.6–1.0.
  d. Spin for 3 min at 1,000 × g and remove the supernatant.
  e. Resuspend the cell pellet in 500 μL of freshly prepared 0.1 M lithium acetate.
  f. Spin for 3 min at 1,000 × g and remove the supernatant.
  g. Boil the single-stranded DNA for 1 min and place it on ice.
  h. Resuspend the cell pellet in Transformation Mix (Table 3).
    ***Note:*** We typically do not exceed 20 μL of total volume for the PCR product and gRNA plasmids, to avoid diluting the transformation mix.
  i. Incubate for 2 h at room temperature (20–24°C).
    **Pause point:** You can store your transformation(s) overnight (up to 16 h) at 4°C at this point.
  j. Incubate for 1 hour in a 42°C water bath.
  k. Spin for 3 min at 1,000 × g and remove the supernatant.
  l. Resuspend the cells in 200 μL of YPD.
  m. Incubate the cells for 3 h at 30°C with shaking.
  n. Plate the cells on agar media selecting for the gRNA plasmid. Use YPD with 200 μg/mL HYG for pML104-HygMX4-URA3 and SD –Ura for pML104-HIS3 and pML104-LEU2.
  o. Incubate the plates for 2–3 days at 30°C.
    ***Note:*** This procedure is a modified version of Gietz’s method.^20^ This extended protocol is often required because many natural yeast isolates are more difficult to transform than standard laboratory strains.
    ***Optional:*** Include a transformation with only the gRNA plasmid and no PCR product. Expect to see very few colonies due to the toxicity of continuous introduction of DNA breaks in the absence of a repair template.^19^
3. Confirm deletion of the auxotrophic marker gene.
  a. Streak colonies from the transformation plate onto YPD to obtain single colonies.
  b. Streak single colonies from the YPD plate onto appropriate selective media to test for the absence of the deleted gene and spontaneous loss of the gRNA plasmid.
    ***Note:*** If needed, 5-FOA can be used to counter-select against *URA3*-containing plasmids. In our experience, this is generally not needed as the majority of yeast colonies will have spontaneously lost the gRNA plasmids after 3 days of growth on YPD.
  c. Perform colony PCR (see step 1) to confirm the absence of the deleted gene. Successful gene deletion will result in a detectable size shift on an agarose gel.
4. Repeat steps 2–3 to consecutively delete additional genes, until the desired genotype is obtained.

**Table 1:** Colony PCR master mix

Reagent	Amount
H_2_O	14.00 μL
Phusion GC buffer (5×, NEB)	5.00 μL
dNTP mixture (10 mM)	0.50 μL
Forward primer (10 μM)	1.25 μL
Reverse primer (10 μM)	1.25 μL
Cell lysate	2.50 μL
Phusion high-fidelity DNA polymerase (NEB)	0.50 μL

**Table 2:** PCR cycling conditions

Steps	Temperature	Time	Cycles
Initial denaturation	98°C	3 min	1
Denaturation	98°C	10 sec	35
Annealing	55°C	30 sec	
Extension	72°C	1 min	
Final extension	72°C	5 min	1
Hold	10°C	hold	1

**Table 3:** Yeast transformation master mix

Reagent	Amount
PCR product	500–1,000 ng
gRNA plasmid	100–500 ng
10 mg/mL single-stranded DNA	5 μL
50% PEG	150 μL
1 M Lithium acetate	29 μL
DMSO	16 μL

## Key resources table

**Table:** 

REAGENT or RESOURCE	SOURCE	IDENTIFIER
**Chemicals, peptides, and recombinant proteins**		

Adenine sulphate	Formedium	Cat#DOC0229
Agar	Formedium	Cat#AGA03
Agarose	Thermo Fisher	Cat#BP160-500
L-Alanine	Formedium	Cat#DOC0104
Ammonium sulphate	Sigma-Aldrich	Cat# A4915
L-Arginine	Formedium	Cat#DOC0108
L-Asparagine monohydrate	Formedium	Cat#DOC0116
L-Aspartic acid	Formedium	Cat#DOC0120
L-Canavanine sulfate salt (CAN)	Sigma-Aldrich	Cat#C9758
L-Cysteine	Formedium	Cat#DOC0124
S-(2-Aminoethyl)-L-cysteine hydrochloride (thialysine, LYP)	Sigma-Aldrich	Cat#A2636
Dextrose	Thermo Fisher	Cat#BP350-1
dNTP Mix (10 mM each)	Thermo Fisher	Cat#18427013
Dimethyl sulfoxide (DMSO)	Sigma-Aldrich	Cat#D8418
Ethylenediaminetetraacetic acid (EDTA)	Sigma-Aldrich	Cat#E9884-100G
5-Fluoroorotic acid monohydrate (5-FOA)	Formedium	Cat#5FOA10-10
G-418 disulphate (G418)	Formedium	Cat#108321-42-2
L-Glutamic acid	Formedium	Cat#DOC0136
L-Glutamic acid monosodium (monosodium glutamate, MSG)	Sigma-Aldrich	Cat#G5889
L-Glutamine	Formedium	Cat#DOC0132
L-Glycine	Formedium	Cat#DOC0140
L-Histidine	Formedium	Cat#DOC0144
Hydrochloric acid 37% (HCl)	Sigma-Aldrich	Cat#258148-100ML
Hygromycin B (HYG)	Formedium	Cat#HYG0500
Inositol	Formedium	Cat#DOC0200
L-Isoleucine	Formedium	Cat#DOC0152
L-Leucine	Formedium	Cat#DOC0156
Lithium acetate	Thermo Fisher	Cat#A17921
L-Lysine	Formedium	Cat#DOC0160
D-(+)-Mannose	Sigma-Aldrich	Cat#63580
L-Methionine	Formedium	Cat#DOC0168
Nourseothricin (NAT)	Jena Bioscience	Cat#AB-102XL
Para-aminobenzoic acid	Formedium	Cat#DOC0204
Peptone	Formedium	Cat#PEP02
L-Phenylalanine	Formedium	Cat#DOC0172
Phusion high-fidelity DNA polymerase	New England Biolabs	Cat#M0530S
Polyethylene glycol 4,000 (PEG)	Thermo Fisher	Cat#A16151
Potassium acetate	Thermo Fisher	Cat#P171500
L-Proline	Formedium	Cat#DOC0176
L-Serine	Formedium	Cat#DOC0180
Single-stranded DNA	Sigma-Aldrich	Cat#D7656
L-Threonine	Formedium	Cat#DOC0184
Tris(hydroxymethyl)aminomethane (Tris)	Thermo Fisher	Cat#17926
Triton X-100	Sigma-Aldrich	Cat#X100-5ML
L-Tryptophan	Formedium	Cat#DOC0188
L-Tyrosine	Formedium	Cat#DOC0192
Uracil	Formedium	Cat#DOC0213
L-Valine	Formedium	Cat#DOC0196
Yeast extract	Formedium	Cat#YEA02
Yeast nitrogen base without amino acids and without ammonium sulphate	Formedium	Cat#CYN0502
Zymolase 100 T *Anthrobacter luteus*	Amsbio	Cat#120493-1

**Experimental models: Organisms/strains**		

TS-allele-on-plasmid collection with SGA markers (inducible gene deletion mutant collection): *MATα xxxΔ::KlLEU2_natR(Cterm) can1Δ::STE2pr-SpHIS5 lyp1Δ0 hoΔ::natMX4 his3Δ1 leu2Δ0 ura3Δ0* [*xxx-ts_natR(Nterm), AgSTE3pr-hphR, URA3*] and *MATα xxxΔ::natR_kanR(Cterm) can1Δ::SpHIS5 lyp1Δ::STE3pr-LEU2 his3Δ1 leu2Δ0 ura3Δ0* [*xxx-ts_kanR(Nterm), AgMFA2pr-hphR, URA3*]	Batté et al.^1^	N/A
BY4741 (*MAT****a****his3Δ1 leu2Δ0 ura3Δ0 met15Δ0*)	Euroscarf	Y00000
DMA809 (*MAT****a****hoΔ:kanMX4 his3Δ1 leu2Δ0 ura3Δ0 met15Δ0*)	Euroscarf	Y03925

**Oligonucleotides**		

URA3_upstream_F: AGGGAAGACAAGCAACGAAA	Batté et al.^1^	N/A
URA3_downstream_R: TCCAGCCCATATCCAACTTC	Batté et al.^1^	N/A
HIS3_upstream_F: TGCACGGTCCTGTTCCCTAGCA	Batté et al.^1^	N/A
HIS3_downstream_R: GCACTTGTTCGCTCAGTTCAGCCA	Batté et al.^1^	N/A
LEU2_upstream_F: GGTCTAAGGCGCCTGATTCA	Batté et al.^1^	N/A
LEU2_downstream_R: TCGCATTATCCTCGGGTTCA	Batté et al.^1^	N/A

**Recombinant DNA**		

pML104-HygMX4-URA3-2	Batté et al.^1^	Addgene #232885
pML104-HygMX4-URA3-3	Batté et al.^1^	Addgene #232886
pML104-HIS3-2	Batté et al.^1^	Addgene #232881
pML104-HIS3-3	Batté et al.^1^	Addgene #232882
pML104-LEU2-2	Batté et al.^1^	Addgene #232883
pML104-LEU2-3	Batté et al.^1^	Addgene #232894

**Software and algorithms**		

Cell Profiler	Carpenter et al.^21^	https://cellprofiler.org
Gitter	Wagih et al.^22^	https://omarwagih.github.io/gitter/

**Other**		

BioMatrix colony processing robot	S&P robotics Inc.	BM5-SC1

**Alternatives:** All listed chemicals can also be bought from other suppliers.

## Materials and equipment

### G418, 1,000× stock solution

Prepare a 200 mg/mL stock solution of G418 in distilled water. Filter-sterilize the stock solution and store it at 4°C for up to six months. Cool media to ∼60°C before adding G418 stock solution. The final concentration should be 200 μg/mL (50 μg/mL for sporulation media).

### Nourseothricin, 1,000× stock solution

Prepare a 100 mg/mL stock solution of nourseothricin (NAT) in distilled water. Filter-sterilize the stock solution and store it at 4°C for up to six months. Cool media to ∼60°C before adding NAT stock solution. The final concentration should be 100 μg/mL.

### L-canavanine, 2,000× stock solution

Prepare a 100 mg/mL stock solution of L-canavanine (CAN) in distilled water. Filter-sterilize the stock solution and store it at 4°C for up to six months. Cool media to ∼60°C before adding CAN stock solution. The final concentration should be 50 μg/mL.

### Thialysine, 2,000× stock solution

Prepare a 100 mg/mL stock solution of thialysine (LYP) in distilled water. Filter-sterilize the stock solution and store it at 4°C for up to six months. Cool media to ∼60°C before adding LYP stock solution. The final concentration should be 50 μg/mL.

**Table:** YPD media

Reagent	Final concentration	Amount
Agar	2% (w/v)	20 g
Yeast extract	1% (w/v)	10 g
Peptone	2% (w/v)	20 g
Adenine	120 mg/L	120 mg
Dextrose 40% (w/v) in H_2_O	2% (w/v)	50 mL
H_2_O	N/A	950 mL
**Total**	**N/A**	**1 L**

N/A, Not applicable.

***Note:*** Weigh the agar, yeast extract, peptone, and adenine, add distilled water and a stir-bar, and autoclave the media. Prepare a 40% dextrose (w/v) solution and autoclave separately. Add 50 mL of 40% dextrose to the autoclaved media to reach a final concentration of 2% (w/v) dextrose. Allow media to cool to ∼60°C before adding small molecules. Store the plates in plastic bags at room temperature (20–24°C). Plates containing small molecules should be stored at 4°C.

**Table:** Amino acid mix for Synthetic Defined (SD) media

Reagent	Final concentration	Amount
Adenine	0.109 g/L	1.5 g
Inositol	0.072 g/L	1.0 g
L-Alanine	0.072 g/L	1.0 g
L-Arginine	0.072 g/L	1.0 g
L-Asparagine	0.072 g/L	1.0 g
L-Aspartic acid	0.072 g/L	1.0 g
L-Cysteine	0.072 g/L	1.0 g
L-Glutamic acid	0.072 g/L	1.0 g
L-Glutamine	0.072 g/L	1.0 g
L-Glycine	0.072 g/L	1.0 g
L-Histidine	0.072 g/L	1.0 g
L-Isoleucine	0.072 g/L	1.0 g
L-Leucine	0.362 g/L	5.0 g
L-Lysine	0.072 g/L	1.0 g
L-Methionine	0.072 g/L	1.0 g
L-Phenylalanine	0.072 g/L	1.0 g
L-Proline	0.072 g/L	1.0 g
L-Serine	0.072 g/L	1.0 g
L-Threonine	0.072 g/L	1.0 g
L-Tryptophan	0.072 g/L	1.0 g
L-Tyrosine	0.072 g/L	1.0 g
L-Valine	0.072 g/L	1.0 g
Para-aminobenzoic acid	0.007 g/L	0.1 g
Uracil	0.072 g/L	1.0 g
**Total**	**2 g/L**	**27.6 g**

***Note:*** Drop-out mix is prepared by omitting the appropriate amino acid(s) from this recipe. Use 2 g of mixed amino acids per liter of media. Store the amino acid mix in a tightly closed container, shielded from light, at room temperature (20–24°C).

**Alternatives:** Dissolve the mixture of amino acids at 20 g/L in distilled water to make a 10× stock solution and filter sterilize. Stock solutions can be stored at room temperature (20–24°C), shielded from light, and added to autoclaved media.

**Table:** SD media

Reagent	Final concentration	Amount
Agar	2% (w/v)	20 g
Amino acid drop-out mix	2.0 g/L	2.0 g
Ammonium sulfate	5.0 g/L	5.0 g
Yeast nitrogen base without ammonium sulfate	1.9 g/L	1.9 g
Dextrose 40% (w/v) in H_2_O	2% (w/v)	50 mL
H_2_O	N/A	950 mL
**Total**	**N/A**	**1 L**

N/A, Not applicable.

***Note:*** For 1 L of media, autoclave the agar separately in 475 mL distilled water. Dissolve the amino acid drop-out mix, ammonium sulfate, and yeast nitrogen base in the remaining 475 mL distilled water and autoclave. After sterilization, combine the two solutions and add 50 mL of 40% dextrose. Allow media to cool to ∼60°C before adding small molecules. Autoclaving the agar and the other ingredients separately increases agar stiffness, which is essential for robotic pinning. Store the plates in plastic bags at room temperature (20–24°C). Plates containing small molecules should be stored at 4°C.

***Note:*** To prepare media containing 5-FOA, add 1 g of 5-FOA powder directly to 1 L of autoclaved media cooled to ∼60°C.

**CRITICAL:** Because ammonium sulfate interferes with G418 selection, replace the ammonium sulfate with 1 g/L monosodium glutamate for SD media containing G418.

**Table:** Enriched sporulation media (SPO)

Reagent	Final concentration	Amount
Potassium acetate	1% (w/v)	10 g
Agar	2% (w/v)	20 g
G418 (200 mg/mL)	50 μg/mL	0.25 mL
Yeast extract	1% (w/v)	1.0 g
Dextrose	0.5 g/L	0.5 g
L-Histidine	12.5 mg/L	12.5 mg
L-Leucine	62.5 mg/L	62.5 mg
L-Lysine	12.5 mg/L	12.5 mg
Uracil	12.5 mg/L	12.5 mg
**Total**	**N/A**	**1 L**

N/A, Not applicable.

***Note:*** Weigh the ingredients, add distilled water and a stir-bar, and autoclave the media. In addition to ingredients that are commonly found in sporulation media (potassium acetate and agar), this recipe also contains yeast extract, amino acids, and glucose. These additional nutrients allow cells to complete several cell cycles before sporulation, ensuring that a sufficient number of cells is present on the sporulation plates for effective robotic pinning. G418 is added to the media after sterilization, to prevent potential contamination. Store media in plastic bags at 4°C.

## Step-by-step method details

### Isolating haploid segregant pools lacking an essential gene

**Timing: 4 weeks**

**Figure 2:**
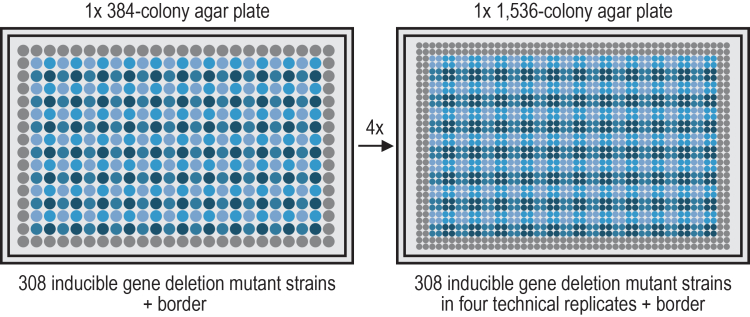
Creating 1,536-format plates The inducible gene deletion mutant collection is shipped and maintained on agar plates containing 384 colonies. Before performing the crosses, each 384-format plate is pinned into 1,536-format, containing four colonies of each strain that are used as technical replicates. Gray colonies represent wild-type border strains; blue colonies represent inducible gene deletion mutants.

This section outlines the procedure for isolating segregant populations in which a gene that is essential in S288C has been deleted, using a protocol that is based on synthetic genetic array analysis (Figure 1).^9^ The inducible gene deletion mutant collection used for this protocol is available upon request from our laboratory and is shipped on agar plates containing 384 colonies each. The collection contains strains with two different genotypes that are maintained on separate plates, referred to as *LEU2*-marked (*MATα xxxΔ::KlLEU2_natR(Cterm) can1Δ::STE2pr-SpHIS5 lyp1Δ0 hoΔ::natMX4 his3Δ1 leu2Δ0 ura3Δ0* [*xxx-ts_natR(Nterm)*, *AgSTE3pr-hphR*, *URA3*]; two plates) and *natR*-marked (*MATα xxxΔ::natR_kanR(Cterm) can1Δ::SpHIS5 lyp1Δ::STE3pr-LEU2 his3Δ1 leu2Δ0 ura3Δ0* [*xxx-ts_kanR(Nterm)*, *AgMFA2pr-hphR*, *URA3*]; five plates) strains. Before crossing this collection to a natural yeast isolate of interest, each 384-format plate is pinned into a 1,536-format plate, containing four colonies per strain (Figure 2). These technical replicates help eliminate false positive cases of cell growth in the isolated segregant pools, which can arise due to spontaneous mutations in the counter-selectable *URA3* marker that prevent removal of the complementing plasmid.

***Note:*** All pinning steps are performed using a BioMatrix colony processing robot (S&P robotics) following standard procedures. Alternatively, a Singer RoToR benchtop robot can be used, or strains can be pinned manually (see Baryshnikova et al.^23^ for details).

1. Prepare agar plates for each step according to the recipes in the materials and equipment section.

**CRITICAL:** Ensure that agar plates are consistent in dryness and thickness, to minimize variation in colony size.

2. Convert each 384-colony inducible gene deletion mutant plate into a 1,536-colony plate containing four colonies per mutant strain (Figure 2).
  a. Pin each 384-colony inducible gene deletion mutant plate four times onto one agar plate with the appropriate selective medium (*LEU2*-marked strains: SD –Ura/Leu, *natR*-marked strains SD –Ura +NAT).
  b. Incubate for 2 days at 26°C.

***Note:*** To prevent integration of the plasmid into the genome, it is important to select for both the essential gene deletion allele (–Leu or NAT) and the plasmid carrying the essential gene (–Ura).

***Note:*** Because the plasmids carry a TS allele of the essential gene, most steps with strains carrying the plasmid are performed at 26°C to ensure proper functioning of the essential gene.

3. Expand the inducible gene deletion mutant collection in 1,536-format.
  a. Pin each inducible gene deletion mutant plate in 1,536-format onto 1–4 agar plate(s) with the appropriate selective media (*LEU2*-marked strains: SD –Ura/Leu, *natR*-marked strains SD –Ura +NAT).
  b. Incubate for 2 days at 26°C.

***Note:*** Each inducible gene deletion mutant plate in 1,536-format contains enough cells for mating with up to four different natural yeast isolates. Expand the collection as needed based on the number of yeast isolates that will be screened by repeating step 3 until the desired number of inducible gene deletion mutant plates is obtained.

4. Prepare lawns of yeast isolates for mating with the inducible gene deletion mutant plates.
  a. Grow the yeast isolate(s) and a S288C control strain (for example, DMA809) overnight (12–16 h) in 3 mL of YPD +G418 +5% mannose at 30°C.
  b. The next day, spread ∼700 μL of overnight culture evenly on a YPD +G418 +5% mannose agar plate.
  c. Incubate for 1 day at 30°C.

***Note:*** One lawn is generally sufficient for mating with four plates of the inducible gene deletion mutant collection. Prepare the necessary number of lawns to conduct all crosses.

***Note:*** Some yeast isolates are difficult to replicate with a pinning tool because they aggregate (flocculate) strongly. Adding mannose to the media reduces aggregation and facilitates pinning. Because aggregation is affected by nutrient availability and cell age, using fresh cultures and lawns will generally improve pinning.

5. Mate the natural yeast isolate with the inducible gene deletion mutant collection.
  a. Pin the yeast isolate from the lawn onto a YPD plate.
  b. Pin the inducible gene deletion mutant collection on top of the natural yeast cells.
  c. Incubate for 1 day at room temperature (20–24°C).

**CRITICAL:** Wetting the pins before pinning from the lawn improves the transfer of yeast cells.

6. Select diploid hybrids using the *kanMX4* cassette of the natural isolate and the *URA3* and *LEU2* or *natR* markers of the inducible gene deletion strain.
  a. Pin crosses of *LEU2*-marked strains onto SD(MSG) –Ura/Leu +G418, and crosses of *natR*-marked strains onto SD(MSG) –Ura +NAT/G418).
  b. Incubate for 2 days at 30°C.
    ***Note:*** Incubating the diploid selection plates at 30°C increases selection against haploid inducible gene deletion mutants that are temperature sensitive.
  c. Pin the diploid selection plates of the *natR*-marked strains from SD(MSG) –Ura +NAT/G418 onto SD(MSG) –Ura +NAT/G418 again.
  d. Incubate for 2 days at 30°C.
    ***Note:*** This extra diploid selection step further depletes any parental *natR*-marked inducible gene deletion mutant strains. In step 8, the *STE3pr-LEU2* cassette present in these strains is used to select *MATα* progeny*.* However, because the parental inducible gene deletion mutant strains have the same mating type, the parental strains can also grow on the media used in step 8, leading to background growth. This extra selection step is not needed for crosses with *LEU2*-marked strains, as for these strains *STE2pr-SpHIS5* is used in step 8 to isolate *MAT****a*** progeny, and this marker is not expressed in the parental *MATα* strains.
7. Sporulate the obtained diploids.
  a. Pin the diploids onto enriched sporulation media.
  b. Incubate for 5–7 days at 22°C.
8. Select haploid segregant progeny.
  a. Pin the sporulated cells onto SD –Arg/Lys/His/Ura +CAN/LYP for *LEU2*-marked strains and SD –Arg/Lys/His/Ura/Leu +CAN/LYP for *natR*-marked strains to select haploid segregant progeny.
  b. Incubate for 3 days at 26°C.

***Note:*** Each colony in this step represents a pool of ∼60,000 independent segregants. The *STE2pr-SpHIS5* cassette selects for *MAT****a*** meiotic progeny in crosses with *LEU2*-marked strains, while the *STE3pr-LEU2* cassette selects for *MATα* meiotic progeny in crosses with *natR*-marked strains. Canavanine (CAN) and thialysine (LYP) are toxic to cells carrying wild-type alleles of *CAN1* and *LYP1*, thereby selecting against any diploid cells that have not sporulated.

9. Select haploid segregant progeny expressing the *kanMX4* cassette.
  a. Pin the haploid progeny onto SD(MSG) –Arg/Lys/His/Ura/Leu +CAN/LYP/G418 to isolate haploid segregant pools expressing *kanMX4.*
  b. Incubate for 2 days at 26°C.

***Note:*** From this step onwards, the same media are used for crosses of *LEU2*-marked and *natR*-marked strains.

10. Select haploid segregant progeny expressing the *kanMX4* and *natR* cassettes.
  a. Pin the haploid *kanMX4*-expressing cells onto SD(MSG) –Arg/Lys/His/Ura/Leu +CAN/LYP/G418/NAT to isolate segregants expressing both *kanMX4* and *natR.*
  b. Incubate for 2 days at 26°C.
  c. Image the plates.
11. Induce loss of the essential gene.
  a. Pin the isolated haploid progeny from step 10 onto SD(MSG) –Arg/Lys/His/Leu +CAN/LYP/G418/NAT/5-FOA media to counter-select against the plasmid and thus induce loss of the essential gene.
  b. Incubate for 2 days at 30°C.
  c. Pin the counter-selection plates onto SD(MSG) –Arg/Lys/His/Leu +CAN/LYP/G418/NAT/5-FOA again to further counter-select against the plasmid.
  d. Incubate for 2 days at 30°C.
  e. Image the plates.

**Figure 3:**
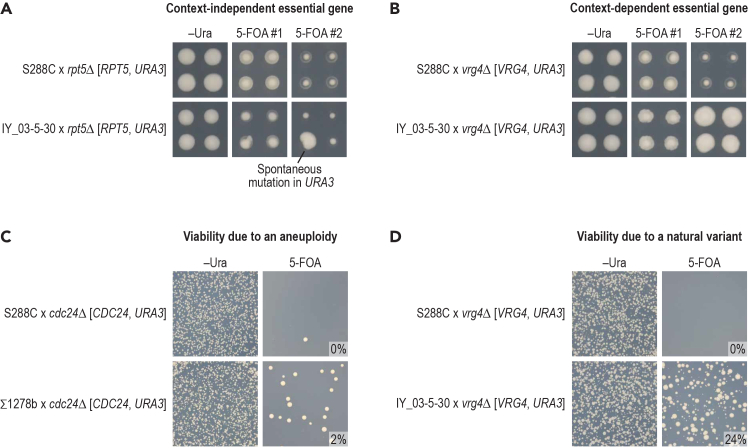
Examples of expected outcomes (A and B) Example images of colonies obtained in steps 10 and 11 of the protocol. *rpt5Δ* [*RPT5*, *URA3*] and *vrg4Δ* [*VRG4*, *URA3*] strains in the reference background were crossed to both S288C and a natural yeast isolate. Each cross was performed in quadruplicate, with each colony containing an independently generated pool of ∼60,000 different haploid progeny. Colony growth is shown in the presence (–Ura, step 10) or absence (5-FOA, step 11) of the plasmid carrying the essential gene. A small “colony” is always visible, even when none of the cells are viable, due to the transfer of dead cells during the pinning. (C and D) Example images of plates obtained in step 20, in case of viability of the essential gene deletion mutant due to either a spontaneous aneuploidy that provides an extra copy of the essential gene (C) or due to natural variants that bypass the requirement for the gene (D). *cdc24Δ* [*CDC24*, *URA3*] and *vrg4Δ* [*VRG4*, *URA3*] strains in the reference background were crossed to both S288C and a natural yeast isolate. Growth of haploid single-colony progeny was determined in the presence (–Ura) and absence (5-FOA) of the plasmid carrying the essential gene. The percentage of colonies that were obtained on 5-FOA media compared with –Ura media is indicated. IY_03-5-30 = IY_03-5-30-1-1-1.

***Note:*** These steps are performed at 30°C because the TS allele is no longer present.

**CRITICAL:** Two sequential pinning steps on 5-FOA are needed to reduce background growth (Figures 3A and 3B).

### Identifying candidate context-dependent essential genes

**Timing: 1 week**

These steps describe how to quantify colony sizes and identify candidate context-dependent essential genes.

12. In R, run gitter^22^ to quantify colony sizes on plate images from steps 10 and 11.
  a. Install and load gitter.
    if (!requireNamespace(“BiocManager”, quietly = TRUE))
      install.packages(“BiocManager”)
    BiocManager::install(“EBImage”)
    library(EBImage)
    if (!requireNamespace(“devtools”, quietly = TRUE))
      install.packages(“devtools”)
    devtools::install_github(“omarwagih/gitter”)
    library(gitter)
  b. Run the following code to quantify colony sizes.
    gitter.batch(files, file.reference, plate.format=1536)
    # files is a directory containing images to be processed
    # file.reference is the path to a reference image
    **CRITICAL:** Because most queried genes will still be essential in the segregants, most mutants will die on 5-FOA media, which complicates automated colony detection. It is thus important to provide gitter with a reference image of a 1,536-format plate with mostly viable colonies, to ensure proper colony detection on 5-FOA plates. A plate from step 10 can be used for this.
    ***Note:*** For each processed plate, two output files are saved to your working directory: an image showing the detected colonies in white, background in black, and colony detection boundaries in red, and a table with the quantified colony sizes in pixels.
    **CRITICAL:** Always check all output images to ensure colonies are correctly detected.
13. Normalize raw colony size measurements of 5-FOA plates (step 11) using the median colony size of the segregant progeny before 5-FOA selection (step 10) to correct for differences in fitness between natural isolates.
14. Perform a Grubbs’ test to identify and filter outlier colonies that occur when one of the technical replicates of a cross carries a spontaneous mutation in *URA3* that causes 5-FOA resistance (Figure 3A).
15. Exclude mutants for which all four technical replicates are alive in the presence of 5-FOA in the S288C control cross.

***Note:*** We recommend considering mutants with a mean normalized fitness > 0.25 in the S288C cross to be alive and to remove these from further consideration.

16. Compare the normalized fitness of the haploid progeny in the presence of 5-FOA between the S288C control cross and the natural yeast isolate crosses and perform a one-sided Welch’s *t* test to determine the statistical significance of observed differences.

***Note:*** We recommend considering genes nonessential in segregant progeny if more than 50% of the strains deleted for that gene had a mean normalized fitness that was at least 0.25 higher in the segregant progeny than in the corresponding S288C control progeny, and the associated *p* value was < 0.02. These cut-offs may differ between experiments and require careful inspection of the data. We recommend validating candidate context-dependent essential genes using an independent assay (steps 17–22).

**CRITICAL:** Examine whether candidate context-dependent essential genes are strongly enriched on specific chromosomes. In some cases, segregant pools may grow on 5-FOA media because an extra copy of the essential gene is present on an aneuploid chromosome that was either inherited from the parental natural isolate or acquired during the screen. Most segregant pools of essential genes located on an aneuploid chromosome will show growth on 5-FOA. Cases of viability of essential gene deletion mutants caused by spontaneous aneuploidies can be distinguished experimentally from cases of context-dependent essentiality caused by natural variants (steps 17–22).

### Validating context-dependent essential genes

**Timing: 2 weeks**

These steps describe experimental procedures for validating candidate context-dependent essential genes identified in step 16.

17. Resuspend a small amount (about one-tenth of a yeast colony) of sporulated cells from the cross of interest in 1 mL of sterile water. Sporulated cells can be obtained from the sporulation plates of step 7.

**Alternatives:** Repeat the cross between the inducible mutant strain of interest and the natural isolate and sporulate the resulting diploids.

***Note:*** Also prepare a suspension of sporulated cells from an S288C control strain crossed to the same inducible gene deletion mutant.

18. Plate 200 μL of resuspended cells in parallel onto two agar plates that select for haploid spores carrying the essential gene deletion allele: one plate that selects for the plasmid carrying the essential gene (SD(MSG) –Arg/Lys/His/Ura/Leu +CAN/LYP/G418/NAT) and a second plate that selects against the plasmid (SD(MSG) –Arg/Lys/His/Leu +CAN/LYP/G418/NAT/5-FOA).
19. Incubate for 3–4 days at 26^°^C.
20. Image the plates.
21. Count the number of colonies per plate. This can be automated using Cell Profiler.^21^
22. Compare the number of colonies obtained in the presence (–Ura media) and the absence (5-FOA media) of the essential gene.

**CRITICAL:** Aim to obtain ∼100–1,000 colonies on media selecting for the essential gene. This range allows for confident detection of a reduction in colony number on media selecting against the essential gene, and ensures clear colony separation for effective automated image analysis.

***Note:*** Cases of context-dependent essentiality driven by one to three modifier variants will result in 12.5% to 50% viable progeny in the absence of the essential gene, relative to progeny carrying the essential gene. In contrast, spontaneous aneuploidies that provide an extra copy of the essential gene lead to 1–3% viable progeny (Figures 3C and 3D).^1^ We therefore consider a gene to be nonessential in a genetic background when at least 10% of the colonies that grow in the presence of the essential gene can also grow in the absence of the gene, a stringent cut-off that allows confident identification of cases of context-dependent essentiality that are driven by modifier variants.

***Note:*** The segregant pools generated by this procedure can subsequently be used to map the genes and genomic alterations underlying context-dependent essentiality. This can be done by whole-genome sequencing of the segregant pools obtained in the presence and the absence of the essential gene and comparing variant allele frequencies in the two populations.^1^

## Expected outcomes

The described protocol enables the identification of genes that are essential in S288C, but vary in essentiality in other yeast isolates. Because the vast majority of genes that are essential in S288C will be essential across genetic backgrounds, most haploid segregant pools will fail to form colonies after counter-selecting against the essential gene (Figure 3A). In rare cases, the natural yeast isolate carries a variant that can bypass the need for the essential gene, and segregants carrying this variant can survive loss of the essential gene and grow on 5-FOA media (Figure 3B).

Colony growth on 5-FOA media, however, can also result from mutations, aneuploidies, or structural variants that prevent loss of the essential gene. For example, spontaneous mutations in the *URA3* counter-selectable marker can cause resistance to 5-FOA in the presence of the plasmid carrying the essential gene. Because each cross is performed in multiple technical replicates, such cases can be readily identified and removed (step 14, Figure 3A). Cells may also acquire an aneuploidy that provides an extra copy of the essential gene, masking the effect of plasmid removal. By assessing the viability of hundreds of single-colony progeny from segregant pools in which growth is observed (steps 17–22), cases caused by spontaneous aneuploidies can be distinguished from cases due to natural variation (Figures 3C and 3D).

Using this protocol, we analyzed 18 yeast isolates from various sources and geographic locations, and discovered 39 context-dependent essential genes (∼5% of tested genes).^1^ Multiple context-dependent essential genes were identified in every isolate studied, suggesting that most natural yeast isolates carry variants capable of modifying gene essentiality.

## Limitations

A limitation of this protocol is that it is restricted to identifying context-dependent essentiality for the 763 genes that are present in the inducible gene deletion mutant array. The approach could be expanded to include an additional 342 genes that are classified as essential in the S288C background, but this would require the construction of inducible gene deletion mutants for these genes. In its current format, this protocol cannot be used to query genes that are nonessential in S288C.

This approach is most effective when applied to euploid isolates. Any aneuploidies that are present in the natural isolate will mask potential cases of context-dependent essentiality of genes located on the aneuploid chromosome. Furthermore, because this approach identifies segregant pools that are viable in the absence of an essential gene, it is possible that a combination of both natural and S288C alleles is needed to bypass the essential gene. In our study of 18 natural yeast isolates, however, we did not identify any cases of context-dependent gene essentiality that required the presence of an S288C allele.^1^

## Troubleshooting

### Problem 1

No colonies are obtained after transformation of a natural yeast isolate (related to section before you begin, step 2).

### Potential solution

• Natural yeast isolates are often difficult to transform. To improve transformation efficiency, optimize parameters such as the number of yeast cells (step 2b), the amount of gRNA plasmid and repair template DNA (step 2h), the duration (0–120 min) and temperature (22–30°C) of the room temperature incubation (step 2i), and the duration (20–180 min) and temperature (42–45°C) of the heat-shock (step 2j) for your specific isolate.^20^^,^^24^ Finally, the duration of the optional incubation at 4°C (between step 2i and 2j) may influence transformation efficiency in either direction.
• Pre-treating the cells with 100 mM DTT for 5–20 min may increase the transformation efficiency by increasing membrane permeability.^24^

### Problem 2

No or few diploids are obtained for the natural yeast isolate crosses (related to step 6).

### Potential solution

• A likely cause is inefficient transfer of natural yeast cells from the lawn to the mating plate (step 5). Natural yeast isolates tend to flocculate or aggregate, making pinning more challenging. If adding mannose to the media and wetting the pins before pinning natural yeast strains from a lawn, as described in steps 4 and 5, does not improve transfer, consider optimizing growth conditions. Possible optimizations include adjusting incubation temperature, increasing mannose concentration, or testing other additives. We note that adding G418 to the media can exacerbate flocculation of certain isolates; in such cases, cultures and lawns can be prepared using media without G418. Alternatively, try pinning the natural isolate from liquid culture onto the mating plate.

### Problem 3

Most colonies grow after the second 5-FOA counter-selection step (related to step 11).

### Potential solution

• Repeat media preparation. 5-FOA may have been present at insufficient levels and/or may have degraded over time. Moreover, if uracil was omitted in the media by mistake, plasmid counter-selection using 5-FOA will fail.
• If the inducible gene deletion mutant array was not maintained correctly, it is possible that the TS alleles have integrated into the genome, preventing counter-selection of the plasmid. Always maintain the collection on media selecting for both the essential gene deletion allele and the plasmid.
• Some yeast isolates are resistant to 5-FOA.

### Problem 4

Colonies are not correctly identified by gitter (related to step 12).

### Potential solution

• Include a reference plate image with viable colonies when running the gitter analysis.

## Resource availability

### Lead contact

Further information and requests for resources and reagents should be directed to and will be fulfilled by the lead contact, Jolanda van Leeuwen (jolanda.vanleeuwen@umassmed.edu).

### Technical contact

Technical questions on executing this protocol should be directed to and will be answered by the technical contact, Jolanda van Leeuwen (jolanda.vanleeuwen@umassmed.edu).

### Materials availability

The key resources table lists all yeast strains and plasmids relevant to this protocol and the sources from which they can be obtained. The inducible gene deletion mutant collection is available upon request. UMass Chan Medical School requires material transfer agreements to be completed for international reagent shipments.

### Data and code availability

The data that support this protocol are available in the supplementary data of the original paper.^1^ This protocol does not require specific code.

## Acknowledgments

We thank members of the Van Leeuwen lab for critical reading of the manuscript. This work was supported by a grant from the 10.13039/501100001711Swiss National Science Foundation (10003570 to J.v.L.).

## Author contributions

Writing – original draft, S.v.S; writing – review and editing, J.v.L.

## Declaration of interests

The authors declare no competing interests.
